# Supplementary material for: Probing complex geophysical geometries with chattering dust
Source: Nat Commun. 2020 Oct 19;11:5282. doi: 10.1038/s41467-020-19087-z (PMC7572509; doi:10.1038/s41467-020-19087-z)
Supplement: Supplementary file 2 — Supplementary Information [file 41467_2020_19087_MOESM2_ESM.pdf]

Supplementary Information

Probing Complex Geophysical Geometries with Chattering Dust

Laura J. Pyrak-Nolte, William Braverman, Nicholas J. Nolte and David D. Nolte

Supplementary Note 1.

3D X-ray microscopy set-up to determine bubble size distribution in chattering dust. A single grain of chattering dust is composed of bubbles of compressed carbon dioxide (4.1 MPa) in a sucrose matrix (made by Zeta Espacial, S.A.). A 3D X-Ray Microscope (Zeiss Xradia 510 Versa) was used to acquire 2D projections to perform 3D computed tomography on individual single dust grains to measure the bubble volume probability and spatial distributions, and to determine the number of bubbles in a single grain. The settings used to acquire the X-ray images are give in Supplementary Table 1 along with the pixel resolution (or voxel edge length) for each sample. The 9 single grains to study the number acoustic emissions per grain (samples CD1A-CD6A) were imaged with a 4x magnification to capture the entire grain volume. Sample G was imaged with a 20x magnification for high resolution imaging to view bubbles smaller than those observed with the 4x objective.

Supplementary Table 1. Sample name and 3D X-ray microscope settings that include the energy, power, source distance, detector distance, optical magnification, exposure time, filter, number of projections and binning. The voxel edge length is listed as pixel size. Field of view = pixel size x 1024 for bin size 2.

Sample	Energy (kV) & Power (W)	Source Distance (mm)	Detector Distance (mm)	Pixel Size (micrometer)	Optical Magnification	Exposure Time (Seconds) & Filter	Number of Projections & (Bin size)
CD1A	50 (4)	-40.07	15.19	5.07	4x	3, LE1	1801 (2)
CD2A	50 (4)	-31.07	15.19	4.533	4x	3, LE1	1801 (2)
CD3A	50 (4)	-40.07	15.19	4.891	4x	3, LE1	1801 (2)
CD3B	50 (4)	-35.07	15.19	4.709	4x	3, LE1	1801 (2)
CD4A	50 (4)	-29.51	15.54	4.4206	4x	3, LE1	1801 (2)
CD4B	50 (4)	-59.98	15.14	5.3894	4x	3, LE1	1801 (2)
CD5A	50 (4)	-36.67	15.84	4.7138	4x	3, LE1	1801 (2)
CD5B	50 (4)	-36.03	22.41	4.2003	4x	3, LE1	1801 (2)
CD6A	50 (4)	-40.94	17.80	4.4046	4x	3, LE1	1801 (2)
G	80 (7)	35.0063	18.0024	0.9072	20x	15, Air	3201 (2)

Data analysis approach for X-ray data. Data reconstruction and analyses were performed using Object Research Systems (ORS) DragonFly Pro 4.0 software for reconstruction and multiregion analysis; and Ilastik[1] for segmentation. After reconstruction of the 3D volume in DragonFly, tiff images are exported to Ilastik to perform segmentation to identify the three features of a dust grain: the gas bubbles, the matrix material, and the air surrounding each grain. Using a pixel classification workflow, training was performed to automatically identify the three features based on color/intensity (Gaussian smoothing), Edge detection (Difference of Gaussians with a sigma=3.5 in 2D), and image texture (methods: Structure Tensor Eigenvalues, and Hessian of Gaussian Eigenvalues). iLastik's wrapper method was applied with a size penalty of 0.1 for comparison against the user feature set to ensure optimal segmentation. However, in most cases, a curated user feature set was used. The simple segmentation and

probability maps were exported as 2D tiff images, and then segmentation was performed on the full 3D stack of 2D tiff images.

The segmented images were then analyzed in DragonFly to calculate the volume, surface area, and center of mass (x,y,z positions) for each segmented bubble. Bubbles included in the distributions shown in Figures 1 & 2 in the manuscript and here in Supplementary Figure 1 were limited to those bubbles with volumes $\geq 4\pi r^3/3$ where r = 5.0*pixel size. This requirement ensures diameters with at least 10 pixels.

Figure 1 in the manuscript contains the results from high resolution imaging (20x objective) of a sub-volume from a single dust grain (sample G in Supplementary Table 1). From the 2D projection (Figure 1a) and 3D reconstruction (Supplementary Figure 1b) a range of bubble sizes is observed. Using the interpretation condition (volumes $\geq 4\pi r^3/3$ where r = 5.0*pixel size), only bubbles with radii greater than 4.5 μm were used in the analysis. For this limited field of view, individual bubble volumes ranged from 500 to 10^7 μm^3, with the most probable bubble volume at this resolution being ~10^3 μm^3 (~ 6.2 μm radius for a spherical bubble).

For samples CDA1-CD6A, the entire grain was imaged with a 4x objective. Again, only bubbles with radii $\geq$ 5*pixel resolution (assuming a sphere) were used in the determination of the bubble volume probabilistic distribution. This limits the interpretation of bubble volume to bubbles with radii greater than 21 to 26 μm. The spatial distribution of bubbles is shown in 3D plots of the center of mass for each bubble for samples CD2A & CD6A (Figure 2 in the manuscript), CD3A & CD4A in Supplementary Figures 1c&f. For this field of view, both bimodal (e.g. Figure 2a in manuscript) and non-bimodal (e.g. Figure 2b) probability distributions of bubble volume were

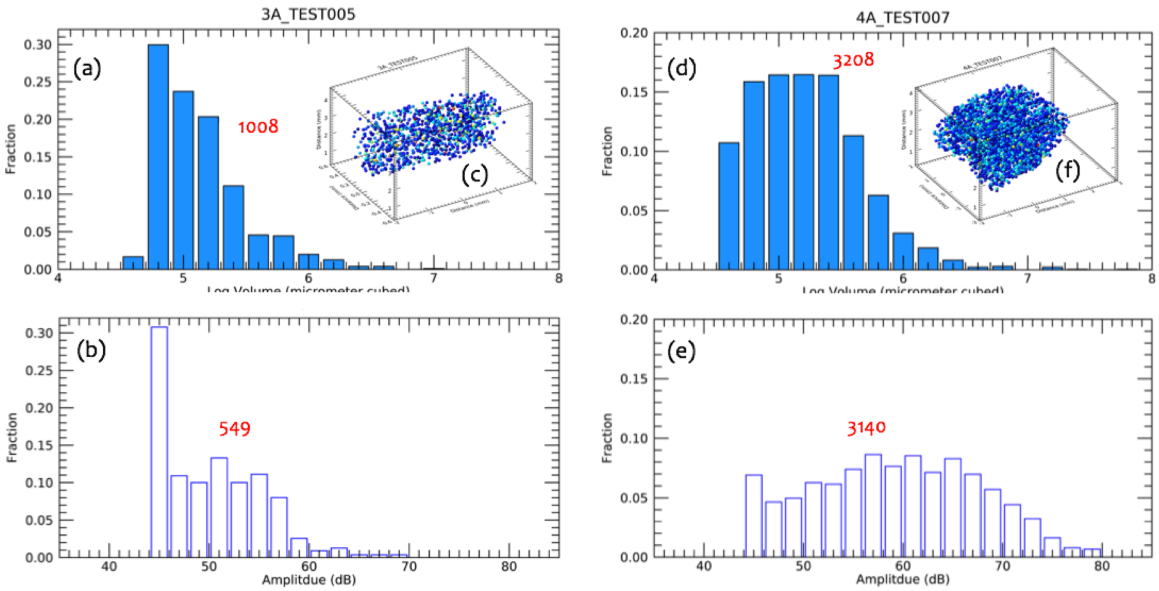

Supplementary Figure 1. (a&d) Probability distribution of bubble volume (in Log_{10}) for single grain samples CD3A and CD4A (see Supplementary Table 1). (b&e) Signal amplitude distribution generated by the bubbles in the grain (number of recorded events from 7 sensor shown in red). (c&f) 3D visualization of the center of mass of the bubbles within each single grain (number in red is number of bubbles)

observed. For the 9 imaged grains, individual bubble volumes ranged between from 3.2 x 10^4 to 2 x 10^7 μm^3, with the most probable bubble volume ~10^5 μm^3 (~ 28.8 μm radius for a spherical

bubble). Supplementary Table 2 contains the number of bubbles and grain volume for each chattering dust grain. From these data, on average there are ~100 gas bubbles/mm^3 (Supplementary Figure 2).

Supplementary Table 2. Sample name, number of bubbles, and estimated grain bulk.

Sample	Bubble Count	Grain Bulk Volume (mm^3)
CD1A	1882	20.54
CD2A	722	13.36
CD3A	1008	11.88
CD3B	1414	7.34
CD4A	3208	16.57
CD4B	977	10.97
CD5A	1547	25.27
CD5B	1342	12.39
CD6A	2091	12.79

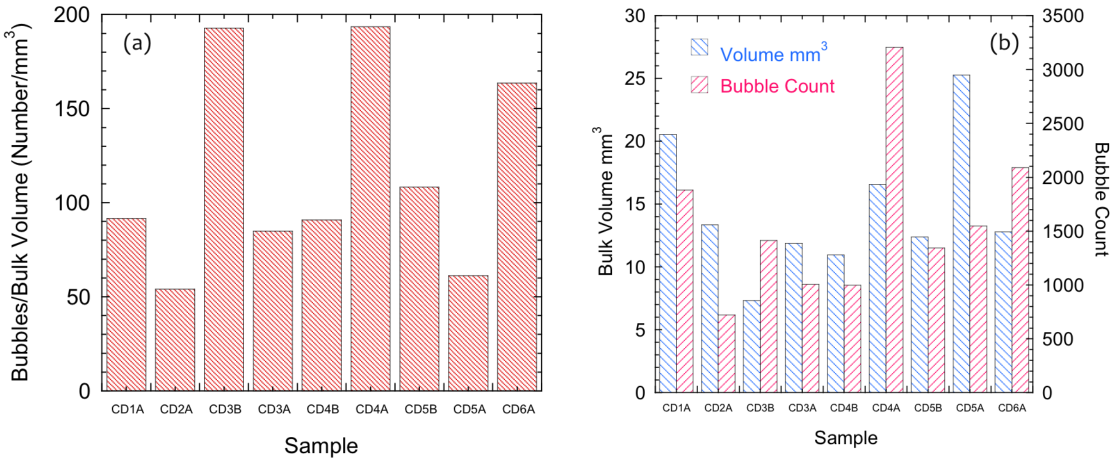

Supplementary Figure 2. (*a*) Bubble density for each grain listed in Supplementary Table 2. (*b*) Comparison of grain bulk volume and bubble count.

Supplementary Note 2.

Methodology for relating acoustic hits to bubble size distributions. After 3-D X-ray microscopy, acoustic emission measurements were performed on the 9 individual chattering dust grains (Supplementary Table 1) to examine the relationship between the number of bubbles and the number of recorded acoustic emission (AE) signals (Supplementary Figure 4), and the log energy probability distribution

Supplementary Table 3. Channel Location on Cuvette (see Supplementary Figure 4)

Sample	x (mm)	y (mm)	z (mm)
Channel 1	40	30	0
Channel 2	65	40	-30
Channel 5	0	20	-65
Channel 6	30	0	-30
Channel 7	40	25	-65

(Supplementary Figure 1b&e and Figure 2b&e in the manuscript). Five broadband AE sensors (Mistra F15A, F-series, Passive Wideband alpha sensor 100-450 kHz) were attached to a prismatic glass cuvette (65 mm x 65 mm 65 mm with wall thickness 2.5 mm) with hot glue. The locations of the sensors on the cube are listed in Supplementary Table 3 for the coordinate system shown in Supplementary Figure 4.

After filling the cuvette with water, the AE sensors were connected to an AE measurement system (24 Channel Mistra Express) through preamplifiers (Mistra 1220-5054, 20/40/60 dB single-ended powered preamplifier) to record signals using Mistra AEWin software. The threshold amplitude for detection was set at 45 dB (with a 60 dB preamplifier setting with 100kHz – 400Khz window) which was determined to eliminate ambient noise for this experimental set-up. An acoustic emission (referred to as a hit) was recorded when the signal amplitude exceeded this threshold. The amplitude of an AE signal is defined as the maximum positive or negative signal excursion during an AE hit. Supplementary Figure 3 shows selected signals emitted from sample grain CD04 during the course of the experiment from the 1st recorded hit at time 17.8273442 seconds to a time of 302.9656898 seconds (Note: the AE system records experimental time out to the 7th decimal place which is 0.1 μs). Each signal is normalized by its maximum positive amplitude to enable visualization of the waveforms. Peak amplitudes varied from 0.177 to 8.91 Volts (45 dB to 79 dB). The AE software converts Volts to dB: dB = 20 log (Vmax/1 μVolt) - (Preamplifier Gain in dB). The AEWin software determined the time and amplitude of every hit and saved the information to a summary line file. In addition, the signals were also recorded (2 MSPS, 100 μs pre-trigger). If more than one event is observed in a single waveform, only one hit per signal was counted (Supplementary Table 4). This potentially leads to an undercounting of AE hits.

Supplementary Table 4. Sample name and hits per channel.

Sample	Channel 1	Channel 2	Channel 5	Channel 6	Channel 7
CD1A	1612	1600	1584	1238	1866
CD2A	494	457	493	493	518
CD3A	630	530	549	505	602
CD3B	911	902	882	797	996
CD4A	3182	3095	3140	2971	3378
CD4B	2863	2865	2851	3033	3015
CD5A	1336	1260	1135	896	1405
CD5B	657	657	677	665	782
CD6A	1786	1777	1706	1706	1908

Supplementary Figure 4 (b) shows the number of AE hits as a function of number of bubbles determined from the 3D X-ray microscopy (see Supplementary Note 1). The number of AE hits increases with an increase in the number of bubbles in a grain. A linear fit to the data shows that fewer events were recorded than the number of bubbles in a sample, which suggests that other events may have been below the selected threshold value for AE detection or occurred within the 100 millisecond window of another event and were not counted. Only for a bubble count > 3000, did the number of events come close to the bubble count.

The amplitude of the recorded hits on all of the channels ranged between 45 to 79 dB (with the lower limit set by the threshold). Figure 2 and Supplementary Figure 1(b&e) show the probability distribution of energy for samples CD2A, CD3A, CD4A and CD6A. Comparison of the

probability distribution functions for the bubble volume and energy of events show relatively similar functional forms.

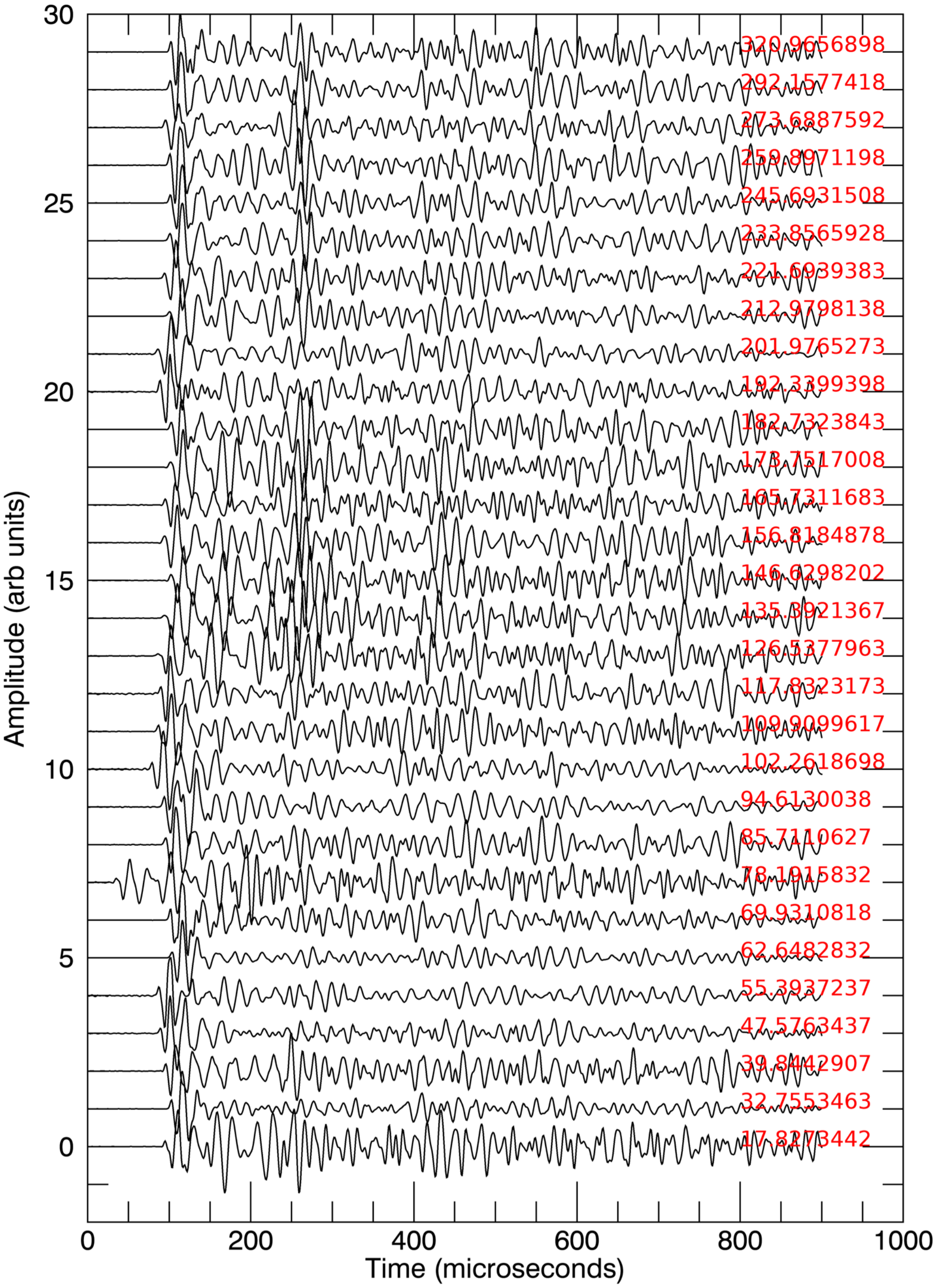

Supplementary Figure 3. Examples of acoustic signals recorded on transducer 6 from dust sample CD04. The red numbers indicate the experimental time of a hit after the initiation of the monitoring (sample CD04).

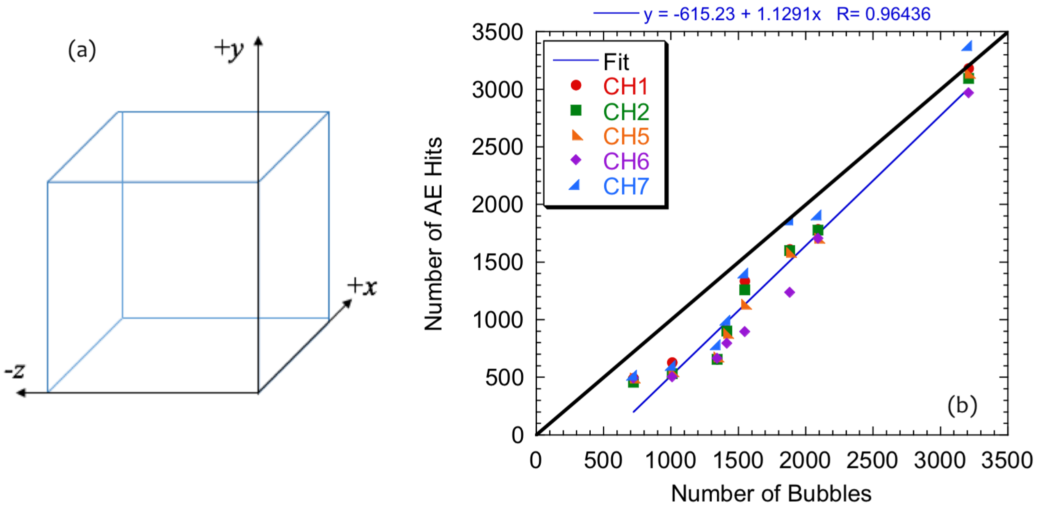

Supplementary Figure 4. (a) Sketch of a glass cuvette with the coordinate system for the transducer locations listed in Supplementary Table 3. (b) Number hits from 9 individual grains of chattering dust as a function of number of bubbles from 3D X-ray microscopy. The X=Y black line is shown for comparison.

Supplementary Note 3.

Methodology for determining explosive nature of source. Experiments were performed to determine if the acoustic wave emitted from a chattering dust grain exhibited an explosive signature with spherical symmetry or asymmetrical behavior associated with shearing or other physical processes. Signals from explosive sources exhibit 1st motion that is in the same direction (i.e. either all positive or all negative excursions after the 1st break) at all sensor locations. A spherical transducer holder was designed and 3D printed to record signals from a single dust grain as it fell under gravity (Supplementary Figure 5). The 13 water-coupled transducers (Olympus Immersion Transducers V303-SU with central frequency 1 MHz) where distributed around the sphere as shown in Supplementary Figure 5a. The sphere had an inner diameter of 96.52 mm with each transducer protruding 17.15 mm into the spherical cavity. The distance between the faces of diametrically opposed transducers was approximately 62.23 mm.

The sphere with transducers was placed on a stand in a tank of water (300 mm x 300 mm x 425 mm). The immersion transducers were connected to the AE measurement system (24 Channel Mistra Express) through preamplifiers (Mistra 1220-5054, 20/40/60 dB single-ended powered preamplifier) to record signals (2 MSps, 200 μs pre-trigger, 2048 points, 0.5 μs/point) using Mistra AEWin software. The threshold amplitude for detection was set at 45 dB (with a 40 dB preamplifier setting with 100kHz – 400Khz window) which was determined to eliminate ambient noise for these experimental conditions. The AE system was initiated and then a single dust grain was released through a hole at the top of the sphere and then fell under gravity. Supplementary Figure 5c compares signals from the 6 transducer pairs with each pair diametrically opposed and at the same depth, and the signal from transducer 1 at the bottom of the set-up. The 1st motion at each sensor shows a positive excursion in amplitude indicating that the source is an explosive source.

Supplementary Note 4.

Methodology for relating dissolution rate and acoustic events. The bottom of a 100 mm diameter petri dish was attached with hot glue to a single AE sensor (Mistra F15A, F-series, Passive Wideband alpha sensor 100-450 kHz) to record the change in acoustic emissions as a particle dissolved in water. For these experiments, the sensor was connected to the previously described AE system with a preamplifier setting of 60 dB and a threshold of 25 dB which was sufficient to eliminate ambient noise for these experimental conditions. The AEWin software recorded the time and amplitude of each hit that was saved to a summary line file. The number of hits from each sample is listed in Supplementary Table 5.

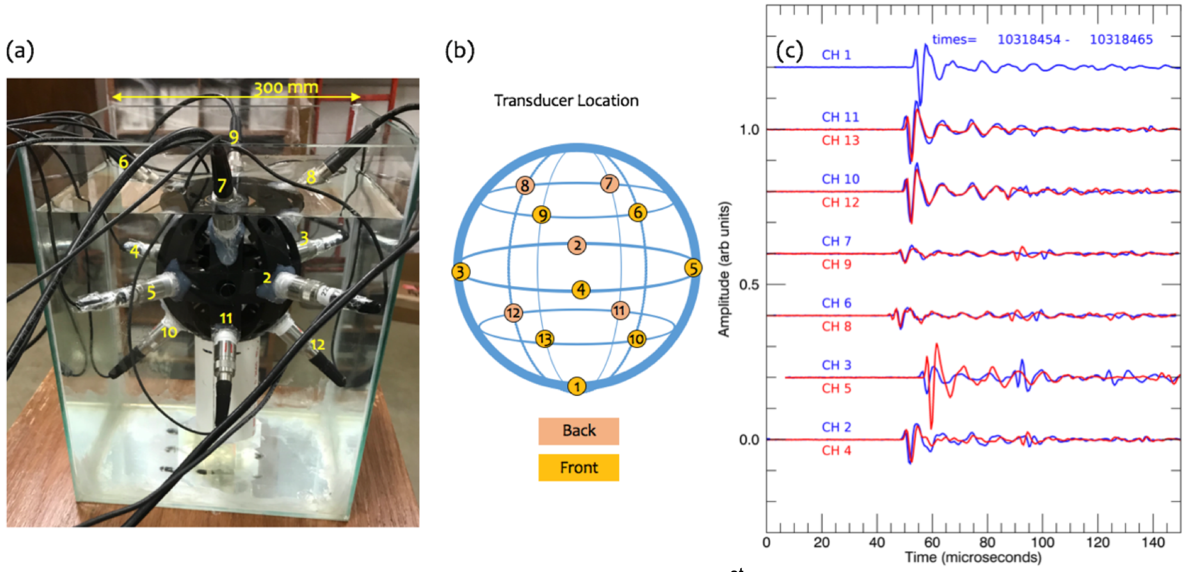

Supplementary Figure 5. (a) Experimental set-up to record 1st motion from single dust grain. (b) Schematic of transducer location in (a). (c) Acoustic signals from a single event coming from signals from transducers at the same depth (vertical direction). Transducer 1 is located on the bottom of the sphere (b) and is in the stand (white tube) in (a).

A custom-built digital imaging system was used to record images of a grain as it dissolved in water (e.g. Figure 3 in manuscript). The system consisted of a Spy camera (Spy camera for Raspberry PI No 1397 from Adafruit) connected to a Raspberry Pi Model B+ with 512MB RAM that captured 3 layered images (red, green and blue (rgb) arrays with dimensions of 2592 pixels x 1944 pixels) at a rate of 1 frames per second. The images were stored as png files directly onto a flash drive. The camera was mounted on a stand at a fixed distance from the sample to obtain images of the grain above the transducer with a resolution of a pixel edge length of ~55 μm. Image analysis was performed with a custom code written in IDL that performed image segmentation by taking the 1st layer of an image and dividing it by the 2nd layer. The location of pixels above a threshold value (see Supplementary Table 5) was used to identify the single dust grain (Figure 3a&d in manuscript blue regions). Pixel counting was used to find the area of a grain[2].

After initiating the AE monitoring and image capture systems, a single dust grain was placed in the water-filled petri dish with tweezers. The water was changed between tests. Of the 12 samples tested, only 3 samples (samples DT13, DT14, DT15 in Supplementary Table 5) disaggregated into 2 or more subparticles over time (e.g. DT14 in Figure 3d in manuscript). The functional form of the time rate of change of the cross-sectional area, $\Delta A/\Delta t$, of the grains differed between particles that exhibited disaggregation and those that did not. Figure 3 in the manuscript shows representative grain area versus time curves for samples DT04 and DT14 along with the number of AE events as a function of time. Note that the number of AE events was binned into 1 second intervals. For DT04 (Figure 3a in manuscript), the image analysis could not resolve the grain for times greater than 146 seconds though acoustic emissions were still occurring. The jump in the curve for DT04 around 50 seconds occurred when the grain rotated and exhibited a different cross-sectional area.

Supplementary Table 5. Threshold value for image segmentation, initial grain area, and number of AE hits for the dissolution study samples.

Sample	Threshold	Initial Area (mm^2)	Number of AE Hits
DT04	1.3	12.71	3325
DT05	1.3	30.69	7887
DT06	1.25	10.18	980
DT07	1.25	12.96	791
DT08	1.12	2.41	178
DT09	1.45	34.36	19008
DT10	1.4	11.89	2601
DT11	1.4	33.09	6805
DT12	1.4	27.14	6003
DT13	1.2	9.56	1035
DT14	1.5	88.33	10970
DT15	1.6	43.19	9195

When a dust grain did not disaggregate (DT04-DT12 Figure 3d in manuscript), the area and number of hits changed linearly in time. For samples DT04-DT12, the average $\Delta A/\Delta t$ of a grain was -0.115 mm^2/s with a standard error of 0.014 mm^2/s. For particles that disaggregated (DT13-DT15), the area and number of hits decreased exponentially with an average decay constant of -0.036 mm^2/s. Single dust grains with a large initial area exhibited a longer duration of acoustic emissions (Supplementary Figure 6a). The correlation between change in dust area and number of AE hits with time was 0.806 (Supplementary Figure 6b).

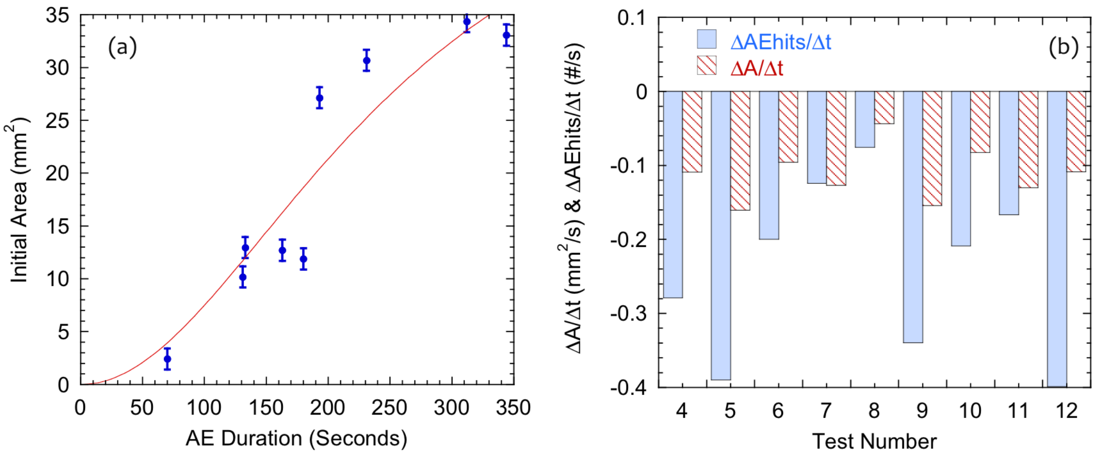

Supplementary Figure 6. (a) Initial area versus the duration of AE events. (b) The time rate of change of the area for single dust grains that did not disaggregate with the time rate of change in number of AE hits.

Supplementary Note 5.

Seeded fracture experimental set-up. The variable-aperture fracture sample (Figure 4 in the manuscript) was an acrylic cast of a fracture in rock acquired from Dr. Timothy Kneasfey at Lawrence Berkeley National Laboratory that was used in a previous study on fluid invasion into an unsaturated fracture[3]. The block measured 0.219 m x 0.307 m with an average thickness of 37.28 mm. Unlike the work of Su et al. [3], no stress or confinement was applied to the sample. 8 AE sensors (Mistra F15A, F-series, Passive Wideband alpha sensor 100-450 kHz) were attached to the sample with hot glue (see Supplementary Table 6 for transducers locations and see Figure 4b&c in manuscript) which were subsequently connected to the previously described an AE monitoring system (see section 2 but a system with only 8 channels and with a preamplifier setting of 40dB and a threshold of 40 dB which in this experiment was sufficient to eliminate ambient noise) to record the full waveform signal (2 MSPs, pretrigger 100 µs, 2048 points). Calibration experiments on location interpretation for the seeded fracture experiment were performed for the transducers location listed in Supplementary Table 6. A drop of water was placed on the rough surface of the fracture and then a single dust grain was released into the drop. Using differences in arrival time differences from among 3 or more transducers with amplitudes greater than or equal to 60 dB, the location interpreted from the AE signals was within $\pm$ 5 mm of the location determined from an image of the dust grain on the fracture surface.

Supplementary Table 6. Positions of AE sensors for the coordinate system for seeded fracture experiment shown in Figure 4 in the manuscript.

AE Sensor	x (cm)	y (cm)
1	15.36	23.37
2	5.39	23.30
3	13.50	19.12
4	8.01	19.81
5	16.28	10.52
6	5.93	9.25
7	12.73	14.14
8	8.11	13.98

The 8 sensors were attached to the top half of the sample. The lower half was seeded with single grains of chattering dust. The upper half was then placed on the lower half, and a gentle pressure was applied to ensure the two fracture surfaces were registered. This process led to crushing of the dust, resulting in several dust fragments in some locations (locations with multiple stars in Figure 4 of the manuscript). Images of the initial seed location were taken for single surface and after registering with the lower half of the sample for comparison. The unsealed fracture sample was placed in a basin (42.9 cm x 35.6 cm x 18.7 cm or ~ 17 L) and inclined 6° above the horizontal. The RaspberryPI image capture system described in section 4 of the Supplement Methods was set-up to record images of the fluid invasion during the course of an experiment. The camera was mounted at an angle a fixed distance from the fracture plane to capture the entire fracture plane, and a light was used to illuminate the fracture plane from below. The basin was white and acted as a light diffuser. Each image was recorded to a jpg file with an image size of 2592 pixels x 1944 pixels x 3 layers at a rate of 1 frame every 2 seconds. Using a constant head (3,731 Pa), water flowed into the basin and was then imbibed into the sample. The pixel edge length was ~160 micrometers. The image capture and AE systems were initiated once the water had imbibed into the lower right corner of the sample (as oriented in Figure 4b&c in the manuscript) and continued throughout the invasion processes (~ 25 minutes). Time syncing between the images and AE events is within +/- 2 seconds. The first hit on the AE system occurred after 10 minutes which was the time for the water to invade to the location of the first dust grain.

Data analysis methods for results from seeded fracture experiments. Image analysis was performed using custom code written in IDL to determine the invasion front as a function of time.

To identify the front, the difference between the 1st layer of an image and the 1st layer of an image from 20 second range prior to the current image was taken. This provides an image of the regions invaded by the fluid in a 20 seconds. Using an image from earlier in the experiment (~56 seconds), a mask of the transducers is created from the 1st layer of the image by finding all pixels less than a threshold of 80 which are then set equal to 1. A dilation algorithm was applied to the mask with a kernel to remove pixel jitter. Then the mask was inverted such that sensor locations were set equal to 0 while all other locations were set equal to 1. Each image was multiplied by the mask, after which a label_region command was used. The front was identified by finding the regions with the highest pixel counts and setting these regions to 1 and discarding the other regions. The color contours shown in Figure 4c in the manuscript represent a 20 second time window on the location of the front with 60 seconds between contours (edge-to-edge).

Interpretation of the location of the chattering dust grain was based on the interpretation by the AEWin software and restricting the interpretation to events recorded by 4-8 sensors with amplitudes greater than 60 dB. To compare the time and location of an AE event with the fluid invasion front, the images were oriented (0.2 degrees counterclockwise rotation to an axis out of the plane) and scaled to match the AE coordinate system. The x- and y- dimensions of the images where scaled to account for the angle between the camera and the fracture plane (5° and 19°, respectively) and with a rigid shift of 5 mm in x and y (which is ~ 3.3% of the total length of the sample). The color of the symbols represents the time of the AE event which uses the same color scale as used to indicate the time of invasion for the contours in Figure 4b in the manuscript.

Supplementary Note 6.

Uniform aperture and variable aperture fractures experimental set-up. Transparent synthetic fractures were fabricated from two acrylic blocks measuring 150 mm x 150 mm x 100 mm (Supplementary Figure 8b) to enable video imaging of the descent of a grain under gravity in a fracture. The aperture was created by separating the blocks by a fixed distance. Uniform apertures of 0.5, 1, 2, 4, 8 and 10 mm were studied. For the variable-aperture fractures, 2 mm thick rubber was cut to create the in-plane variable geometry (Supplementary Figure 8c and Figure 6a-d in the manuscript) and was placed between the two acrylic blocks (Figure 6 in manuscript). 8 AE sensors (Mistra F15A, F-series, Passive Wideband alpha sensor 100-450 kHz) were attached to a sample with hot glue (see Supplementary Table 7 for transducers locations and example signals in Supplementary Figure 7) which were subsequently connected to the previously described AE monitoring system (see section 2 but with a preamplifier setting of 60dB and a threshold of 35 dB which in this experiment was sufficient to eliminate ambient noise) to record the full waveform signal (2 MSPs, pretrigger 100 µs, 2048 points).

Supplementary Table 7. Positions of AE sensors for the coordinate system for the uniform aperture fracture experiments (Supplementary Figure 8b where b is the fracture aperture).

AE Sensor	x (mm)	y (mm)	z (mm)
1	25	-25	100+b/2
2	-25	-25	100+b/2
3	-25	25	100+b/2
4	25	25	100+b/2
6	33	45	-100-b/2
7	-33	45	-100-b/2
8	-33	-42	-100-b/2
9	33	-42	-100-b/2

The RaspberryPI image capture system described in section 4 of the Supplemental Methods was used to video the descent of each grain during each experiment. The camera was mounted a fix distance from the fracture plane to capture the entire plane. The video was recorded as a

file mp4 file with image size 280 pixels by 480 pixels and a frame rate of 29 fps. The imaging and AE measurements were repeated 7-15 times for the uniform and variable fracture geometries.

Interpretation of chattering dust location from video images: For analysis, the videos were converted using Free WMV AVI Converter software to 640 pixel x 480 pixels in with a frame rate of 25 fps, and then exported from QuickTime Player 7 as png images (640 pixel x 480 pixels). The pixel resolution in the images ~470 µm edge length. A custom code was written in IDL to segment the images to identify a dust grain and track the location of the dust as it fell. This was achieved by taking a subimage (50 pixels wide by 315 pixels in length) and then thresholding the image. Using the data cube (x-y-t, where t=time), two arrays were created from the threshold images to observe the lateral and vertical path from 20-500 images (# in sum depends on aperture and speed of descent): (1) a single image from the sum of y-t planes, and (2) a single image from the same data cube but of the x-t plane. These two arrays were then thinned, and the x- and y-locations of the maximum pixel value were found and exported to a text file along with the image time. The speed of descent was determined from a linear fit to the data in the graphing software Kaliedgraph.

Interpretation of chattering dust location from acoustic emission. Custom codes were written in IDL to (1) sort the signals by events where an event is defined as an acoustic emissions recorded by 3 or more AE sensors; (2) perform a Hilbert transform on each signal to determine an arrival time based on the first peak of the Hilbert transform (Supplementary Figure 7); and (3) find the x- and y- positions of the dust from the difference in arrival time using a non-linear Broyden solver. This approach assumes (a) a direct path between the source (dust) and the receiver; (b) that the dust fell in the center of the fracture (z = 0 in Supplementary Figure 8b); and (c) a system velocity of V=2630 m/s where the system includes the acrylic blocks and the water-saturated fracture and is based on the arrival time of the peak of the Hilbert which is later than the first break. The acoustic speed in acrylic is 2730 m/s, for water at 20°C the speed is 1480 m/s and for in solidified hot glue is 1870 m/s. The selected value of V was based on a minimization approach that takes advantage of the dust floating on the surface of the water prior to descending into the fracture. While the dust is floating, it provides a reference or calibration point for determining the velocity of the system.

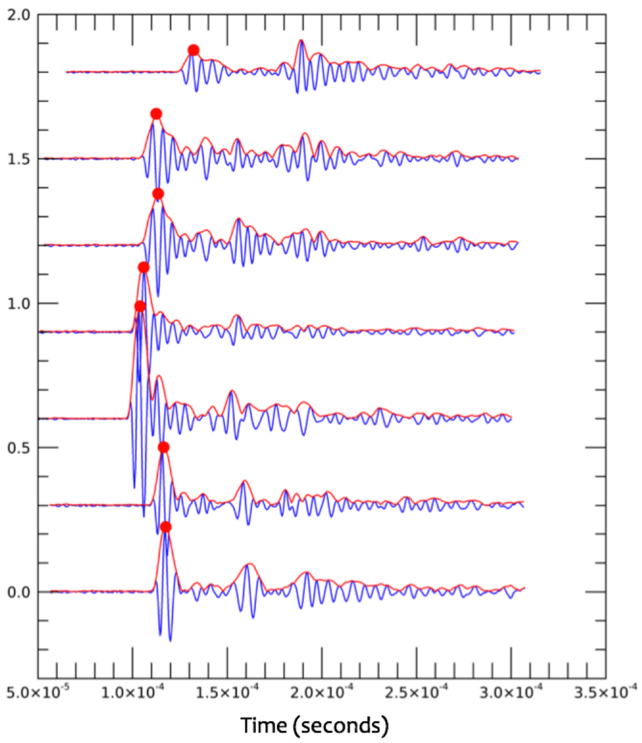

Supplementary Figure 7. Hilbert transform (red lines) of signals (blue lines) from an event. Red dot shows the arrival time used in the analysis.

A dust grain can float or remain at the air-water interface at the top of fracture for two reasons: (1) an initial hydrophobicity that maintains the particle at the air-water interface until it starts to dissolve; and (2) the particle dimension is too large to fit into the fracture aperture. The average float time as a function of aperture is shown in

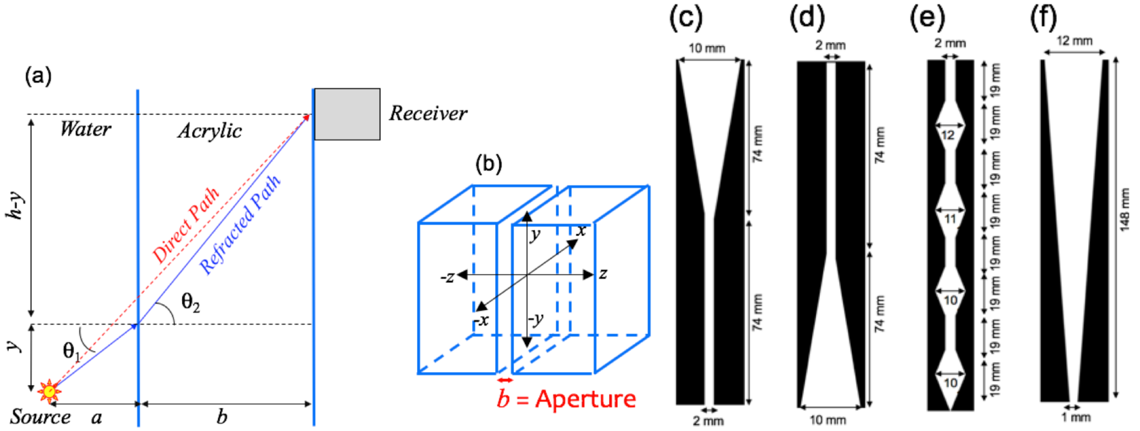

Supplementary Figure 8. (a) 2D schematic of refraction of an acoustic ray path at the interface between water and acrylic. (b) Coordinate system used in the forward modeling with sensor locations given in Supplementary Table 6. (c) Geometric details of the variable aperture fractures (c) “Y”, (d) “Inverted Y”, (e) “Diamond Chain” and (f) ‘Converging Aperture” shown in Figure 6 in the manuscript.

Supplementary Figure 9a. The float times in apertures 4 mm to 10 mm are attributed to reason (1). For apertures < 2mm, the float times are long because the particles were not small enough to fit into the aperture. Assuming that a spherical particle with a radius ~ 2 mm dissolves to 0.4 mm to fit within the 0.5 mm aperture, the time rate of change in cross-sectional area of a grain would need to be $\Delta A/\Delta t \sim -0.101$ mm^2/s (assuming a circular cross-section) for the average float time of 120 seconds (Supplementary Figure 9a). This value of $\Delta A/\Delta t$ is comparable to the value from the dissolution experiment described in section 4 of this supplement ($\Delta A/\Delta t$ -0.115 mm^2/s with a standard error of 0.014 mm^2/s). It is also important to note that dissolution is a function of surface area as dissolution rate decreases with decreasing surface area.

As a particle descends, or is transported through a system, its dimensions change because of dissolution. Descent times, $t_{descent}$, ranged from ~15 to 1.5 seconds for fractures with uniform apertures of 0.5 mm and 10 mm, respectively (with average velocity of descent = $\langle V_{descent} \rangle \sim 10$ mm/s and 100 mm/s). Using the average $\Delta A/\Delta t = -0.115$ mm^2/s, the A of a grain would decrease by ~1.3 mm^2 for the 0.5 mm aperture and by 0.415 mm^2 for the 10 mm aperture fracture or roughly a change in radius, Δr, (assuming a circular cross section) of 0.741 mm and 0.234 mm, respectively. This change in the radius of a grain would affect $V_{descent}$ because the drag from the walls decreases with distance from the wall. An indirect measure of the effect of particle dimensions is shown in Supplementary Figure 9bc&d. Supplementary Figure 9c illustrates the effect of disaggregation on interpreting $V_{descent}$ and also shows the repeatability of $V_{descent}$ and suggests an effect of particle size, with the 3rd particle falling the faster and having been subjected to dissolution the longest. Supplementary Figure 9d examines the effect of volume on $V_{descent}$. The size of a chattering dust grain used in these experiments was measured against a ruler for three orthogonal directions. The $V_{descent}$ interpreted from the video images exhibits a first order dependence that shows a linear increase in velocity with size when the particle is much less than the aperture but decreases in speed as the particle approaches the size of the aperture. This is hypothesized to explain the large variation in velocity observed in the measurements for the 1 mm aperture (Supplementary Figure 9c). Knowledge of the dissolution rate and time enable estimates of changes in aperture.

From the data for the 1 mm aperture fracture (specifically Test013), a velocity was selected by determining the average location of the first 100 points when a dust grain was floating and finding the velocity that yielded the minimum value (i.e. depth y ~ 75 mm in Supplementary Figure 8b). This approach yielded the full possible descent path from 0 to 150 mm depth. Once the velocity was determined, events were located by using a Broyden non-linear solver to fit the *x* & *y* positions of the source and the time to source for sets of transducer combination (in groups of 3). The error bars shown in Figure 5a in the manuscript are based on the averaged location from all of the groups for a given event. The error bars in Figure 5b in the manuscript are from the average of 7-10 tests performed for each aperture. The Broyden approach is related to Newton's method for finding function zeroes, but it has higher efficiency because it calculates the entire Jacobian up front rather than at each iteration. The signal from transducer 1 was chosen arbitrarily as the reference signal. The set of equations solved by the Broyden routine are based on the the following equation:

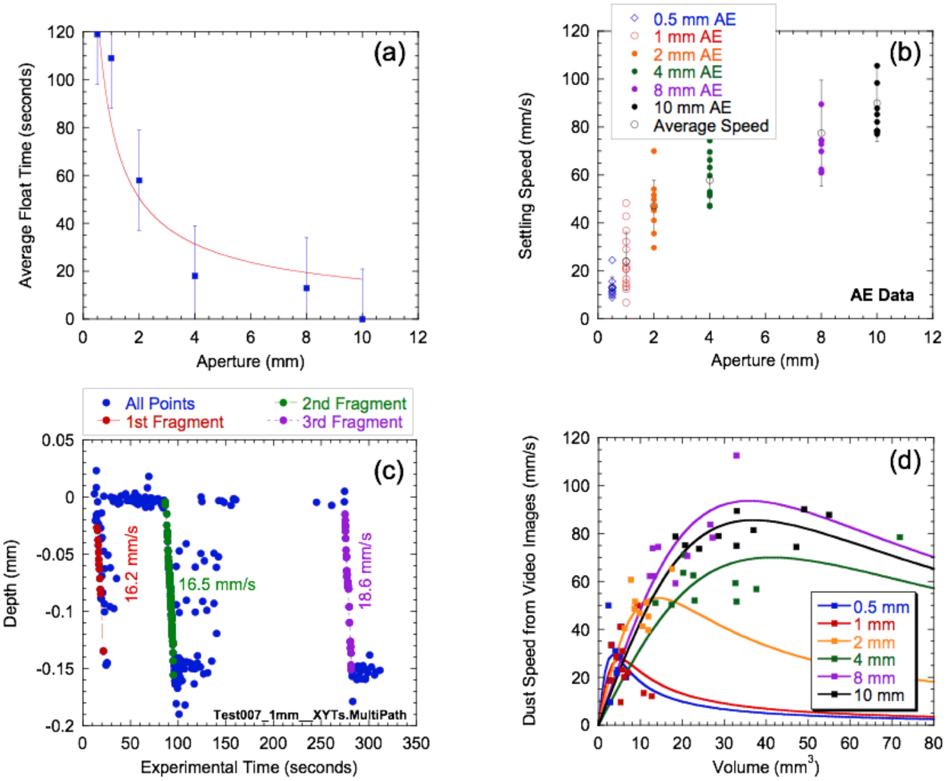

Supplementary Figure 9. (a) Average time that a single dust grain floats before descending into a fracture as a function of fracture aperture. (b) Settling speed for all tests for each aperture as a function of aperture. (c) Interpreted depth as a function of experimental time for a disaggregating particle in a 1 mm aperture fracture with the interpreted $V_{descent}$ for each particle. (d) Dust speed from video image analysis as a function of initial volume of a dust grain.

$$\frac{\sqrt{(x_{ti}-x_s)^2+(y_{ti}-y_s)^2+(z_{ti}-z_s)^2}}{V}-(t_s-\Delta t_i)=0 \qquad \text{(se1)}$$

where t_s is the travel time from the source to transducer 1, the subscript t_i represents transducer "i", *v* is the velocity, and the location is given by *x*, *y*, and *z*. The z location of the source was assumed to be at the center of the fracture plane, z_s=0. The minimum in the value of the first 100

y = 0 locations, i.e., zero crossings, as a function of velocity are used to select V in equation (se1). V= 2630 m/s was used to find the position of the source (x_s, y_s) and t_s for every event during the experiment.

Analysis of interpretation of chattering dust location with and without assuming refraction. A numerical study was performed to determine the error in the interpretation of dust grain settling velocity that can occur from not considering refraction of the emitted acoustic signal as it is transmitted from the source through water and into the acrylic block. A custom IDL code was written to perform forward modeling based on Fermat's principle of path of least time for the refracted path (Supplementary Figure 8*a*). The coordinate system for the simulations is given Supplementary Figure 8*b*, and the sensor locations in Supplementary Table 7.

The fracture wall defines a planar interface between the water and the acrylic matrix. The plane of the wall (150 mm x 150 mm x-y plane in Supplementary Figure 8b) was discretized into 50 μm x 50 μm elements. For each transducer location, the time from the center of the transducer to each wall element, t_{tw}, on the fracture wall was calculated using the speed of sound in acrylic ($V_{acrylic}$ = 2730 m/s) and a path length, $r_{acrylic}$, of

$$r_{acrylic} = \sqrt{(h-y)^2 + b^2} \qquad \text{(se2)}$$

where h is the total height of the fractures (150 mm y-direction). The fracture height was discretized in 1 mm increments. The emission from a dust grain falling under gravity in water was treated as a point source. The travel time for a wave that traveled from the source to the fracture wall, $t_{sw,}$ was calculated using the speed of sound in water (V_{water} = 1480 m/s) and the path length, r_{water}, of

$$r_{water} = \sqrt{y^2 + a^2} \qquad \text{(se3)}$$

where a is the distance of the source to the fracture wall. The travel path was taken as the path of least time between a source and a receiver, namely, when $t_{tw} + t_{sw}$ was a minimum. The minimum

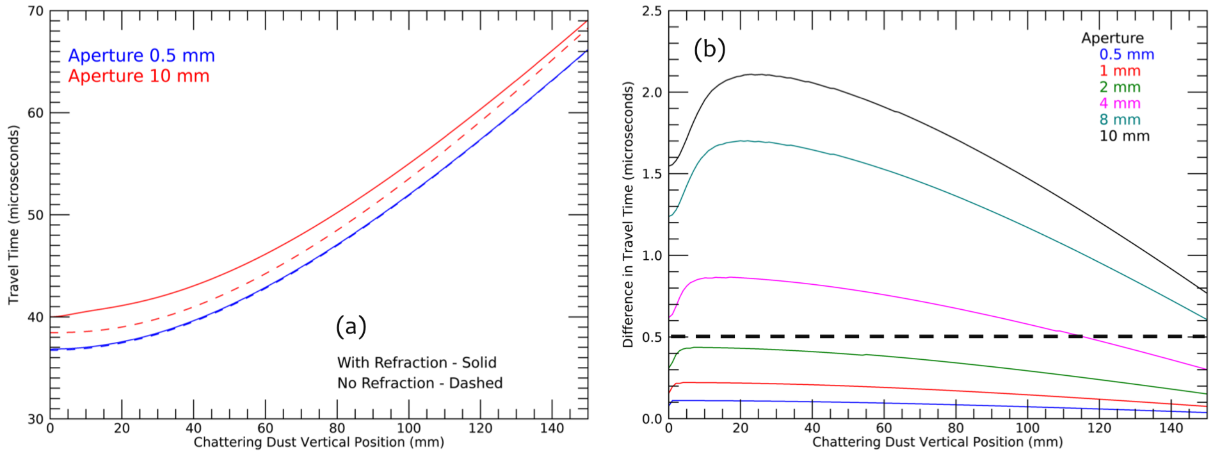

Supplementary Figure 10. (a) Comparison of travel time from dust assuming a no refraction and refraction for apertures of 0.5 mm and 10 mm. (b) Time difference between direct path and refracted path for different aperture fractures.

distance occurs when $\sin \theta_1/\sin \theta_2 = V_{water} / V_{acrylic}$. For comparison, the travel time along the direct path (red path in Supplementary Figure 10a) was also calculated assuming a velocity along the entire ray direct path of a fitted-$V_{acrylic}$ (based on locating the floating dust see Supplementary Note 6) which is the assumption that was used in the analysis of the experimental data. Supplementary Figure 10 provides a comparison of the travel time from a descending source to receiver position 1 (sensor 1 in Supplementary Table 7) for water-filled fractures with an aperture 0.5 mm and 10 mm assuming both that the ray is, and is not, refracted. The difference in travel time between the two ray paths is shown in Supplementary Figure 10b for all simulated and measured apertures. For apertures b = 0.5 to 2 mm, the time difference is below the resolution of AE monitoring system (dashed line). The AE system records signals with 0.5 μs/point. A dt = 0.5 μs in acrylic yields a difference in path length of 1.365 mm. For b > 2 mm, the difference in arrival time increases.

The travel times between each source position and 8 sensor locations (4 on the $z= -0.075$ x-y plane and 4 on the $z=+0.075$ mm x-y plane with the locations used in the experiments, see Supplementary Table 7 & Supplementary Figure 8*b*) were calculated to determine the error in x location and interpretation of the speed of descent of a dust particle. The time differences (assuming sensor 1 as the reference as in the data analysis of the experiments) were used in the Broyden non-linear solver described above to determine the location of the source as it fell along the path (x,z)=(0,0) for y= -0.075 to $+0.075$ mm in 1 mm increments for apertures 0.5, 1, 2, 4, 8, and 10 mm.

As in the experiments, a fitted velocity, fitted-$V_{acrylic}$, was determined that yielded a total travel path of the dust ~150 mm. For a grain falling along the (x,z)=(0,0), Supplementary Tables 8 lists the average and standard deviation in the x location of the dust and the ratio of the speed of descent based on including refraction, V_{dr}, to the speed of descent assuming a direct path, V_{dd}, between the source and receiver, as assumed in the experiments by fitting $V_{acrylic}$ = 2717 m/s for all apertures. The x location deviated from x=0 by 157 μm to 2.6 mm with increasing aperture and resulted in 1-3% under prediction of the speed of descent, $V_{descent}$ (Supplementary Table 8). When $V_{acrylic}$ is interpreted for each aperture (Supplementary Table 9), the under-prediction in $V_{descent}$ decreases and the interpreted x location is off from x=0 by 100 μm to 550 μm with increasing aperture.

In general, deviations in the interpretation of dust location and speed of descent increase with increasing aperture. For the experiments presented here, the error from assuming a direct path in the interpretation of speed of descent is on the order of 1-3% which is smaller than the error from experimental measurements from averaging the location from sensor groups for each event.

Supplementary Table 8. From simulations, the ratio of the speed of descent when including refraction compared to the direct path, average interpreted x location and standard deviation for a range of apertures assuming $V_{acrylic}$ = 2717 m/s for all apertures.

Aperture (mm)	V_{dr}/ V_{dd}	Average x location (mm)	STD in x (mm)
0.5	1.009	0.157	0.184
1	1.010	0.275	0.155
2	1.013	0.544	0.146
4	1.018	1.069	0.262
8	1.028	2.102	0.590
10	1.03	2.601	0.756

Supplementary Table 9. From simulations, the ratio of the speed of descent when including refraction compared to the direct path, average interpreted x location and standard deviation for a range of apertures by fitting $V_{acrylic}$ for each aperture.

Aperture (mm)	Fitted $V_{acrylic}$ (m/s)	V_{dr}/ V_{dd}	Average x location (mm)	STD in x (mm)
0.5	2715	1.010	0.285	0.204
1	2717	1.010	0.275	0.158
2	2720	1.011	0.352	0.100
4	2728	1.011	0.370	0.176
8	2743	1.011	0.501	0.505
10	2751	1.012	0.550	0.657

Supplementary Note 7.

Methodology for transport of chattering dust through inverted T fracture intersection. The intersecting fractures used in the flow experiments were fabricated from 3 acrylic blocks as shown in Figure 6 in the manuscript. The horizontal block (bottom) measured 150 mm x 150 mm x 50 mm in height. The left block measured 100 mm x 150 mm x 150 mm and the right block measured 50 mm x 150 mm x 150 mm. The surfaces of the blocks were smooth except for the bottom face of the right block which exhibited saw-cut roughness. The entire sample was submerged in a water tank (diameter ~ 0.88 m and height ~ 0.66 m). The aperture of the fractures were ~4-5 mm. The top of the vertical fracture was unsealed to enable water to be pumped through the vertical fracture and also the horizontal fracture. The left & right sides (as shown in Figure 6 in the manuscript) each contained 3 ports that could be opened or closed individually during transport. Fluid was pulled or pushed through the horizontal fracture with a 265 Lph water pump attached to one side of the fracture and the other end of the pump open to the water tank.

Supplementary Table 10. Transducer locations for Inverted T fracture intersection.

Transducer	x (mm)	y (mm)	z (mm)
1	38	-133	55
2	-46	-133	72
3	21	-133	105
4	-29	-133	123
5	4	-133	189
6	-29	82	55
7	55	82	72
8	-12	82	105
9	38	82	123
10	4	82	190
11	0	-70	-21
12	-34	-34	-21
13	1	-17	-21
14	-17	17	-21
15	34	34	-21

15 immersion transducers (Olympus Immersion Transducers V303-SU with central frequency 1 MHz) were used to monitor the system. The transducers were connected to AE system through the preamplifiers (see section 2 in this supplement). The threshold amplitude for detection was set at 35 dB (with a 60 dB preamplifier setting with 100kHz – 400Khz window) which was determined to eliminate ambient noise for these experimental conditions. The signals were recorded (10 MSps, with a 100 μs pre-trigger) and saved, along with a summary line file, using Mistra AEWin software. The locations of the transducers are given in Supplementary Table 10 for the coordinate system shown in Figure 6 of the manuscript.

The AE system was initiated prior to the release of a single dust grain in the vertical fracture.

The dust was released through a narrow tube (~ 3 mm diameter) into the vertical fracture by applying a stream of water from the nozzle of a water bottle. This ensured the descent of the particle into the fracture system. The location of release was varied during the experiment. Interpretation of the location of the chattering dust grain was based on the interpretation by the AEWin software and restricting the interpretation to events recorded by 8-15 transducers.

Supplementary Note 8.

Calculation of moment magnitude. The energy, E, released from an average bubble (radius ~ 28 μm) with a pressure of 4.1 MPa is roughly E ~ 0.4 μJ. Assuming E ~ M_o, where M_o is the seismic moment in Nm, the estimated moment magnitude[4], M_w, for a for a single bubble determined from $M_w = (\log_{10} M_o - 9.05)/1.5$ is $M_w \sim -10$.

Supplementary Note 9.

Determination of Current Detection Range. Experiments were performed to determine the maximum detection distance to provide an idea of the scalability of the current chattering dust approach. A single sensor (Mistra R.45 with bandwidth of 5-30 kHz) was affixed to a concrete

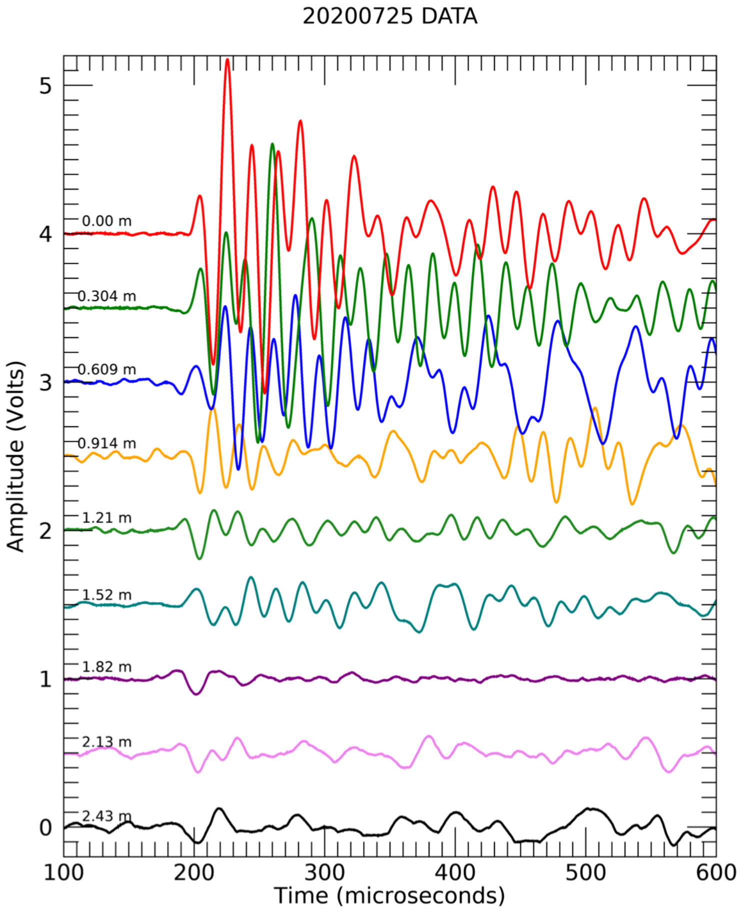

Supplementary Figure 11. AE signals from dust grains placed from adjacent to the transducer (0.00 m red signal) to 2.43 m (black signal) away from the transducers.

floor with hot glue (~ 1 mm in thickness). Distances were marked on the floor in 0.3048 m increments proceeding away from the transducer. The AE sensor was connected to an 8 channel AE measurement system (Mistra Express) through preamplifiers (Mistra BP-SYS, 20/40/60 dB single-ended powered preamplifier) to record signals using Mistra AEWin software. The threshold amplitude for detection was set at 40 dB (with a 60 dB preamplifier setting with 10kHz – 100Khz window) which was determined to eliminate ambient noise for this experimental set-up. The signals were recorded (10 MSps, with a 300 μs pre-trigger) and saved, along with a summary line file, using Mistra AEWin software. After initialization of the AE system, water was placed at the nearest location marked on the floor, and dust grains were placed in the water. AE signals were recorded until the grain dissolved completely. Then the process was repeated every 0.3048 m up to a distance of 2.4 meters. Supplementary Figure 11 shows the signal amplitude at successive distances from the transducer. The detection limit under these conditions is approximately 2 meters on concrete.

Supplementary References

1 Berg, S. et al. ilastik: Interactive machine learning for (bio)imaging analysis. Nature Methods **16**, 1226-1232, doi:https://doi.org/10.1038/s41592-019-0582-9 (2019).
2 Chen, D. Q., Pyrak-Nolte, L. J., Griffin, J. & Giordano, N. J. Measurement of interfacial area per volume for drainage and imbibition. Water Resources Research **43** (2007).
3 Su, G. W., Geller, J. T., Pruess, K. & Wen, F. Experimental studies of water seepage and intermittent flow in unsaturated, rough-walled fractures. Water Resources Research **35**, 1019-1037 (1999).
4. Hanks, T. C. and H. Kanamori, A moment magnitude scale, Journal of Geophysical Research, vol. 84, no B5, 2348-2350 (1979
